# Polymorphisms in the *NSUN1* gene and neuroblastoma risk in Chinese children from Jiangsu province

**DOI:** 10.7150/jca.103097

**Published:** 2025-01-01

**Authors:** Jiaming Chang, Chunlei Zhou, Wei Jia, Haixia Zhou, Tianyou Yang, Zhuorong Zhang, Haiyan Wu, Yan Zou, Jing He

**Affiliations:** 1 Department of Pediatric Surgery, Guangzhou Institute of Pediatrics, Guangdong Provincial Key Laboratory of Research in Structural Birth Defect Disease, Guangzhou Women and Children's Medical Center, Guangzhou Medical University, Guangdong Provincial Clinical Research Center for Child Health, Guangzhou 510623, Guangdong, China.; 2 Department of Pathology, Children's Hospital of Nanjing Medical University, Nanjing 210008, Jiangsu, China.; 3 Department of Hematology, The Key Laboratory of Pediatric Hematology and Oncology Diseases of Wenzhou, The Second Affiliated Hospital and Yuying Children's Hospital of Wenzhou Medical University, Wenzhou 325027, Zhejiang, China.

**Keywords:** neuroblastoma, susceptibility, *NSUN1*, polymorphism, m5C modification

## Abstract

Neuroblastoma is the most prevalent extracranial solid tumor among children and exhibits remarkable heterogeneity. The methylation of cytosine to form 5-methylcytosine (m5C) is the primary type of modification found in DNA and RNA. The NOL1/NOP2/sun (NSUN) family, specifically NSUN1, is responsible for the methylation process and has been shown to play a key role in cell differentiation and cancer development. Nevertheless, the impact of *NSUN1* gene polymorphisms on neuroblastoma risk remains uncertain. Two selected *NSUN1* gene polymorphisms (rs11834074 G>A and rs3764909 C>A) were genotyped via the TaqMan method in a study population consisting of 402 neuroblastoma patients and 473 cancer-free controls. The associations between two selected polymorphisms and neuroblastoma risk were evaluated using odds ratios (ORs) and 95% confidence intervals (CIs). Neither the overall analysis nor the stratification analysis revealed a significant correlation between these two polymorphisms and the risk of neuroblastoma (rs11834074 G>A, AA vs. GG: adjusted OR=0.99, 95% CI=0.58-1.67, *P*=0.964; GA/AA vs. GG: adjusted OR=0.91, 95% CI=0.70-1.19, *P*=0.478; AA vs. GG/GA: adjusted OR=1.04, 95% CI=0.63-1.73, *P*=0.876; while for the rs3764909 C>A polymorphism, AA vs. CC: adjusted OR=1.03, 95% CI=0.66-1.62, *P*=0.901; CA/AA vs. CC: adjusted OR=0.95, 95% CI=0.73-1.24, *P*=0.710; AA vs. CC/CA: adjusted OR=1.07, 95% CI=0.70-1.64, *P*=0.767). Collectively, these findings indicate that the two selected *NSUN1* polymorphisms may not be associated with neuroblastoma susceptibility. However, further studies with larger sample sizes and additional potentially functional polymorphisms are needed to validate these results.

## Introduction

Neuroblastoma, which garners substantial attention, prevails as the foremost extracranial solid tumor afflicting children. This type of tumor accounts for approximately 15% of pediatric tumor-related mortality 1. At present, there is a general consensus that neuroblastoma derives from incompletely developed sympathetic neural precursors 2. The morbidity rate of neuroblastoma in Western countries ranges between 10.1 and 15.0 cases per million children 3, whereas it is 7.72 cases per million among Chinese children aged 0-14 years 4. This type of tumor exhibits a high degree of heterogeneity in terms of both biological and clinical characteristics. The survival rate approaches 90% for low and intermediate-risk patients, whereas it is remains below 40% for high-risk patients 5. Despite undergoing intensive and comprehensive treatment, which includes chemotherapy, surgical intervention, immunization therapy, and radiotherapy, patients with high-risk neuroblastomas still have a poor prognosis and a high recurrence rate 6. Given that early-stage cancer is more likely to have a positive prognosis, the identification of genetic risk factors for neuroblastoma has emerged as a key area of research focus.

Previous research has demonstrated that genetic factors significantly contribute to the tumorigenesis of neuroblastoma. It is widely acknowledged that neuroblastoma patients with amplified *MYCN* oncogenes typically have a poorer prognosis. Additionally, several other significant genetic mutations have been identified in neuroblastoma, including *TERT* 7, *ALK* 8, *ATRX* 9, and *RAS* 10, as well as segmental chromosome aberrations (1p and 11q) 11. Although the aforementioned genetic variations provide valuable insights, they fail to comprehensively elucidate all the risk factors contributing to neuroblastoma.

The high degree of heterogeneity indicates that individual genetic variation plays a crucial role in neuroblastoma. As the most prevalent type of genetic alteration in DNA, single nucleotide polymorphisms (SNPs) constitute the fundamental units for analyzing complex genetic variations 12. Over the past several decades, SNPs have been extensively studied for their correlation with disease susceptibility, tumorigenesis 13, and individual variations in response to therapeutic drugs 14. Consequently, SNPs are regarded as pivotal points bridging genetic research with personalized precision medicine.

5-methylcytosine (m5C) modification has a significant effect on the biological function of RNA, performing a pivotal role in cell differentiation, survival, proliferation, and migration, thereby serving as a cornerstone in these cellular processes 15. Serving as the primary enzymes responsible for m5C RNA methylation, the NOL1/NOP2/sun (NSUN) family utilizes S-adenosylmethionine as a methyl donor to transfer methyl groups, ultimately resulting in the formation of m5C 16. A recent study revealed the essential role of NSUN1 in ribosome biogenesis, rRNA processing, and cellular proliferation 17. NSUN1 is overexpressed in various types of cancer, including colorectal cancer 18, glioma 19, oral carcinoma 20 and ovarian cancer 21. Furthermore, the NSUN1 expression level is correlated with tumor invasiveness, unfavorable prognosis, and responsiveness to treatment 22. The depletion of the *NSUN1* gene not only suppresses the migration, proliferation and invasion of colon cancer cells 23, but also increases the sensitivity of leukemia cells to 5-azacytidine treatment 24. Collectively, these findings establish the *NSUN1* gene as a potential therapeutic target and valuable prognostic indicator. However, the connection between *NSUN1* gene polymorphisms and neuroblastoma susceptibility is still unclear, necessitating further research to clarify the role of *NSUN1* gene variations in neuroblastoma. Most SNPs with regulatory activity are located in the coding region 25, and *NSUN1* gene rs11834074 G>A and rs3764909 C>A polymorphisms are located in the intron region of chromosome 12. The SNPs within introns has the potential to cause irregular splicing of precursor mRNAs, thereby impacting gene function 26. To date, no studies have reported that the rs11834074 G>A and rs3764909 C>A polymorphisms have any effect on the activity or expression of *NSUN1*.

To the best of our knowledge, no research has investigated the role of the *NSUN1* gene in neuroblastoma, let alone explored the possible association between *NSUN1* gene polymorphisms and neuroblastoma predisposition. Given the significant role of the *NSUN1* gene in cancer, a comprehensive analysis and consideration of the potential impact of its SNPs on neuroblastoma are warranted. With the aim of examining this hypothesis, we conducted a case-control study aimed at identifying potentially functional polymorphisms (rs11834074 G>A, and rs3764909 C>A) of *NSUN1*and carried out a comprehensive analysis to elucidate the impact of *NSUN1* gene polymorphisms on neuroblastoma susceptibility among Chinese children.

## Materials and Methods

### Study subjects

This study included a total of 402 neuroblastoma patients and 473 cancer-free individuals as controls (**Table S1**) 27, 28. The neuroblastoma patients were recruited from the Children's Hospital of Nanjing Medical University from January 30, 2008 to November 2, 2021. The samples were obtained from the hospital sample library or surgical biopsy specimens. All patients were newly diagnosed with neuroblastoma. The healthy control group included age-matched, healthy children who underwent routine health examinations at same hospital during the same period. Prior to commencing the research, this study was approved by the Institutional Review Committee of Nanjing Medical University Children's Hospital (approval number: 202112141-1), thereby guaranteeing adherence to ethical standards and regulatory requirements. The authors strictly adhered to the directives provided by the committee, assuming complete responsibility for all aspects of the investigation, thus ultimately guaranteeing accuracy, reliability, and reproducibility of the results.

### Polymorphism selection and genotyping

Using the dbSNP database and SNPinfo software, we identified two potentially functional polymorphism sites in the *NSUN1* gene (rs11834074 G>A, and rs3764909 C>A). Both of these two polymorphisms were located in the intron region of *NSUN1* gene, and are predicted to be transcription factor binding sites. SNPs in low linkage disequilibrium (LD) with other SNPs were selected by applying an R^2^ threshold of less than 0.8, and the R^2^ was 0.596 between the two selected polymorphisms. Using a DNA Kit (TianGen Biotech Co. Ltd. DP332), genomic DNA was extracted from the blood or tumor tissue samples provided by each study subject. The genotyping of *NSUN1* polymorphisms (rs11834074 G>A, and rs3764909 C>A) was carried out using TaqMan real-time PCR method (Kit from TianGen Biotech Co. Ltd. EP324) 29-32. To ensure genotyping accuracy, 10% of the samples were randomly chosen for regenotyping, which confirmed complete agreement between the two genotyping datasets.

### Statistical analysis

The χ^2^ test was employed to assess the demographic features and genotype frequencies in both neuroblastoma patients and healthy controls. The χ^2^ goodness-of-fit test was applied to evaluate the adherence to the Hardy-Weinberg equilibrium (HWE) within the control group. To establish the association between *NSUN1* polymorphisms and neuroblastoma susceptibility, odds ratios (ORs) and 95% confidence intervals (CIs) were computed. Adjustments for age and sex were made by computing adjusted ORs and their corresponding 95% CIs and performing multivariate logistic regression across various patient subgroups. Our analysis comprehensively evaluated polymorphic sites using recessive, dominant, and additive genetic models. Furthermore, relative risk odds ratios, 95% CIs, and their corresponding *P* values were determined. All the statistical analyses were carried out using SAS software version 9.4 (SAS Institute, Cary, NC), with a statistical significance threshold set at *P* values under 0.05.

## Results

### Associations of *NSUN1* polymorphisms with neuroblastoma susceptibility

In this study, genotyping of two SNPs (rs11834074 G>A, and rs3764909 C>A) was effectively accomplished in 402 neuroblastoma patients and 473 cancer-free controls. Specifically, the neuroblastoma patients presented with an average age of 40.99 ± 35.49 months, whereas the healthy control group presented with an average age of 40.88 ± 29.76 months. The distributions of these SNP frequencies in the control group followed the HWE (HWE=0.068 for rs11834074 G>A; HWE=0.293 for rs3764909 C>A). After age (*P*=0.100) and sex (*P*=0.987) were considered, the results revealed no significant associations between the two polymorphisms of the *NSUN1* gene and neuroblastoma susceptibility (for the rs11834074 G>A polymorphism, GA vs. GG: adjusted OR=0.90, 95% CI=0.68-1.18,* P*=0.434; AA vs. GG: adjusted OR=0.99, 95% CI=0.58-1.67, *P*=0.964; GA/AA vs. GG: adjusted OR=0.91, 95% CI=0.70-1.19, *P*=0.478; AA vs. GG/GA: adjusted OR=1.04, 95% CI=0.63-1.73, *P*=0.876, while for the rs3764909 C>A polymorphism, CA vs. CC: adjusted OR=0.93, 95% CI=0.70-1.24, *P*=0.629; AA vs. CC: adjusted OR=1.03, 95% CI=0.66-1.62, *P*=0.901; CA/AA vs.CC: adjusted OR=0.95, 95% CI=0.73-1.24, *P*=0.710; AA vs. CC/CA: adjusted OR=1.07, 95% CI=0.70-1.64, *P*=0.767) in the single-locus analysis (**Table 1**).

### Stratification analysis of selected SNPs

To assess the potential associations between the selected SNPs and neuroblastoma risk across diverse subgroups, a stratified analysis was performed taking into account factors such as age, sex, tumor origin site, and clinical stage (**Table 2**). However, the findings failed to establish any significant correlation between the individual *NSUN1* polymorphisms (rs11834074 G>A, and rs3764909 C>A) and neuroblastoma susceptibility among the examined subgroups. Consistent findings were similarly observed in the subsequent combined analyses.

## Discussion

Neuroblastoma is the predominant solid tumor in children, noted for its highly remarkable heterogeneity 1. The prognosis and treatment options for neuroblastoma patients are intricately dependent on their risk classification, resulting in varying outcomes and management approaches based on the specific risk group 33. The low-risk type may spontaneously regress without any intervention. Conversely, high-risk neuroblastoma carries a considerable risk of recurrence and mortality, even after undergoing a series of intensive treatments 34. A thorough analysis of the genetic factors contributing to neuroblastoma risk is critical for an early diagnosis and personalized precision medicine.

An increasing number of studies have shown that the occurrence of cancer is an interactive phenotype triggered by a combination of gene mutations and epigenetic mechanisms 35, 36. As a type of epigenetic modification, RNA modifications have been shown to undergo aberrant changes in cancer 37. Recent research has demonstrated that methylation regulates the development of neuroblastoma 38, 39. Given the significant role of m5C modification in tumorigenesis, our research focused primarily on m5C RNA modification genes. Previously, our group reported a statistically significant correlation between a variation in the m5C demethylase gene *ALKBH1*, specifically the rs2267755 polymorphism, and a reduction in neuroblastoma risk 40. In addition, our earlier study revealed that functional genetic variations in m5C demethylase genes, specifically *TET1* and *TET2*, confer an increased risk of neuroblastoma 41, 42. Importantly, the polymorphism in the *NSUN2* gene, rs13181449 C>T, diminishes neuroblastoma susceptibility 27. Collectively, these findings suggest that variations in m5C modification-related genes, particularly polymorphisms in *NSUN* family genes, are correlated with neuroblastoma risk. As a pivotal member of the *NSUN* gene family, evaluating the impact of *NSUN1* gene polymorphisms on susceptibility to neuroblastoma is essential. Therefore, we carried out a case-control study aimed at examining the correlation between variations in the m5C demethylase gene *NSUN1* and neuroblastoma risk among children in East China.

Our study included 402 neuroblastoma patients and 473 cancer-free controls. To the best of our knowledge, no other studies have explored the relationship between *NSUN1* gene polymorphisms and neuroblastoma risk. The principal objective of this study was to exhaustively examine the associations between genetic variations in the m5C demethylase gene *NSUN1* and the neuroblastoma susceptibility among the pediatric population in China. However, the findings revealed that neither of the two selected SNPs, rs11834074 G>A and rs3764909 C>A, manifested a statistically significant association with the risk of neuroblastoma.

m5C, a widely researched type of RNA modification type, commonly occurs in both coding and noncoding RNAs 15. The equilibrium in gene modifications is maintained by the coordinated action of m5C-binding proteins (ALYREF and YBX1), methyltransferases (NSUN family, TRDMT family, and DNMT2), demethylases such as ALKBH1 and members of the TET family. m5C functions as a crucial regulator of genomic stability and gene expression in DNA. Only recently, the diverse biological functions of m5C, including RNA translation, RNA export, RNA fragmentation, and ribosome composition, have been revealed 43. Previous studies have suggested that m5C modification plays an extensive role in many processes, including cellular differentiation, cellular signaling, tissue development, and cancer pathogenesis 44. On the other hand, the role of m5C RNA modification in cancer disease is also indispensable 45, 46. Notably, the *NSUN* family regulates m5C modifications across a wide range of RNA types, including mRNAs, tRNAs, rRNAs, mitochondrial RNAs, enhancer RNAs, vault RNAs, noncoding RNAs, and even viral RNAs 47. *NSUN1* mediates ribosome synthesis and processing by binding to rRNA and facilitating the process of ribosome metabolism 48. In addition, *NSUN1* has been shown to bind to mRNA and regulate the cell cycle 49. Furthermore, *NSUN*1 has been implicated in both tumorigenesis and cell differentiation processes 50.

Nonetheless, in our present case-control investigation, we did not detect any associations between the chosen SNPs under consideration and neuroblastoma susceptibility. Based on our assessment, it appears unlikely that the two selected SNPs (rs11834074 G>A and rs3764909 C>A) have any effect on the expression or function of the *NSUN*1 gene. Hence, these SNPs are unlikely to alter neuroblastoma susceptibility. Despite our inability to establish a direct correlation between *NSUN1* SNPs and neuroblastoma susceptibility, our latest research revealed significant associations between neuroblastoma risk and other genes involved in m5C methylation. To date, N6-methyladenosine (m6A) and m5C have been widely recognized as the most dominant types of RNA methylation. Preliminary research has indicated that m6A modification has a significant effect on neuroblastoma 51. A review hypothesized that RNA function could be jointly regulated by m6A and m5C modifications, presenting a compelling focal point for future research, especially when contemplated in conjunction with our previous findings 44.

Nevertheless, this study still has the following shortcomings. First and foremost, the generalizability of the conclusions is restricted as this study was conducted solely and exclusively within the premises of a single center, with all participants recruited from a single hospital. In addition, the genetic profile may vary depending on the ethnic group of the population evaluated and other environmental factors associated with disease progression are not considered in these case-control association studies. Additionally, the moderate sample size in this study imposes constraints on the statistical power of the analysis. Conducting further research utilizing expanded sample size holds the potential to generate varying conclusions and significantly augment the credibility and reliability of statistical results. Finally, owing to our assessment being confined solely to two specific polymorphisms within the *NSUN1* gene, there remains a possibility that we may have neglected other significant SNPs that could affect neuroblastoma susceptibility.

Overall, our exhaustive research findings unequivocally demonstrate that neither the *NSUN1* gene rs11834074 G>A polymorphism nor the rs3764909 C>A polymorphism is significantly associated with neuroblastoma susceptibility in the Chinese population. It is worthwhile to pursue further studies that encompass expanded sample sizes and rigorously investigate additional potential functional SNPs.

## Supplementary Material

Supplementary table.

## Figures and Tables

**Table 1:** Associations between *NSUN1* gene polymorphisms and neuroblastoma susceptibility in children from Jiangsu province

Genotype	Cases (N=402)	Controls (N=473)	*P* ^ a^	Crude OR (95% CI)	*P*	Adjusted OR (95% CI) ^b^	*P* ^ b^
rs11834074 G>A (HWE=0.068)							
GG	200 (49.75)	224 (47.36)		1.00		1.00	
GA	172 (42.79)	215 (45.45)		0.90 (0.68-1.18)	0.437	0.90 (0.68-1.18)	0.434
AA	30 (7.46)	34 (7.19)		0.99 (0.58-1.67)	0.965	0.99 (0.58-1.67)	0.964
Additive			0.616	0.95 (0.77-1.17)	0.617	0.95 (0.76-1.17)	0.615
Dominant	202 (50.25)	249 (52.64)	0.480	0.91 (0.70-1.19)	0.480	0.91 (0.70-1.19)	0.478
Recessive	372 (92.54)	439 (91.81)	0.877	1.04 (0.63-1.73)	0.876	1.04 (0.63-1.73)	0.876
rs3764909 C>A (HWE=0.293)							
CC	175 (43.53)	200 (42.28)		1.00		1.00	
CA	182 (45.27)	223 (47.15)		0.93 (0.70-1.24)	0.629	0.93 (0.70-1.24)	0.629
AA	45 (11.19)	50 (10.57)		1.03 (0.66-1.62)	0.902	1.03 (0.66-1.62)	0.901
Additive			0.889	0.99 (0.81-1.21)	0.889	0.99 (0.81-1.21)	0.889
Dominant	227 (56.47)	273 (57.72)	0.710	0.95 (0.73-1.24)	0.710	0.95 (0.73-1.24)	0.710
Recessive	357 (88.81)	423 (89.43)	0.768	1.07 (0.70-1.63)	0.767	1.07 (0.70-1.64)	0.767
Protective genotypes ^c^							
0	5 (1.24)	2 (0.42)		1.00		1.00	
1	235 (58.46)	270 (57.08)		0.35 (0.07-1.81)	0.210	0.35 (0.07-1.82)	0.211
2	162 (40.30)	201 (42.49)		0.32 (0.06-1.68)	0.180	0.32 (0.06-1.69)	0.180
0-1	240 (59.70)	272 (57.51)		1.00		1.00	
2	162 (40.30)	201 (42.49)	0.511	0.91 (0.70-1.20)	0.512	0.91 (0.70-1.20)	0.508

OR, odds ratio; CI, confidence interval; HWE, Hardy-Weinberg equilibrium. ^a^ χ^2^ test for genotype distributions between neuroblastoma cases and cancer-free controls. ^b^ Adjusted for age and gender. ^c^ Protective genotypes were carriers with rs11834074 GA/AA and rs3764909 CC/CA genotypes. 0: rs11834074 GG and rs3764909 AA. 0-1: rs11834074 GG and rs3764909 AA, rs11834074 GA and rs3764909 AA, rs11834074 AA and rs3764909 AA, rs11834074 GG and rs3764909 CC, rs11834074 GG and rs3764909 CA. 1: rs11834074 GA and rs3764909 AA, rs11834074 AA and rs3764909 AA, rs11834074 GG and rs3764909 CC, rs11834074 GG and rs3764909 CA. 2: rs11834074 GA and rs3764909 CC, rs11834074 GA and rs3764909 CA, rs11834074 AA and rs3764909 CC, rs11834074 AA and rs3764909 CA.

**Table 2:** Stratification analysis for the association between *NSUN1* gene polymorphisms and neuroblastoma susceptibility

Variables	rs11834074 (cases/controls)		AOR (95% CI) ^a^	*P* ^a^	rs3764909 (cases/controls)		AOR (95% CI) ^a^	*P* ^a^	Protective genotypes (cases/controls)		AOR (95% CI) ^a^	*P* ^a^
	GG	GA/AA			CC	CA/AA			0-1	2		
Age, month												
≤18	74/71	65/68	0.92 (0.57-1.47)	0.712	62/59	77/80	0.91 (0.57-1.47)	0.710	89/88	50/51	0.97 (0.59-1.58)	0.897
>18	126/153	137/181	0.92 (0.67-1.27)	0.609	113/141	150/193	0.97 (0.70-1.34)	0.853	151/184	112/150	0.91 (0.66-1.26)	0.569
Gender												
Females	94/110	97/115	0.99 (0.67-1.46)	0.952	87/94	104/131	0.86 (0.58-1.27)	0.439	111/132	80/93	1.03 (0.69-1.52)	0.902
Males	106/114	105/134	0.84 (0.58-1.22)	0.360	88/106	123/142	1.04 (0.72-1.52)	0.819	129/140	82/108	0.82 (0.57-1.20)	0.308
Sites of origin												
Adrenal gland	46/224	47/249	0.91 (0.58-1.42)	0.678	40/200	53/273	0.98 (0.62-1.53)	0.917	57/272	36/201	0.84 (0.53-1.33)	0.448
Retroperitoneal	85/224	82/249	0.87 (0.61-1.24)	0.438	76/200	91/273	0.88 (0.62-1.25)	0.468	101/272	66/201	0.89 (0.62-1.27)	0.510
Mediastinum	56/224	64/249	1.03 (0.69-1.54)	0.897	46/200	74/273	1.18 (0.78-1.78)	0.430	68/272	52/201	1.03 (0.69-1.55)	0.874
Others	10/224	8/249	0.73 (0.28-1.88)	0.510	10/200	8/273	0.58 (0.22-1.49)	0.255	11/272	7/201	0.88 (0.33-2.31)	0.793
Clinical stages												
I+II+4s	80/224	93/249	1.04 (0.73-1.48)	0.831	70/200	103/273	1.10 (0.77-1.57)	0.605	102/272	71/201	0.92 (0.65-1.32)	0.664
III+IV	89/224	74/249	0.74 (0.52-1.06)	0.103	78/200	85/273	0.80 (0.56-1.15)	0.228	100/272	63/201	0.84 (0.59-1.22)	0.365

OR, odds ratio; CI, confidence interval. ^a^ Adjusted for age and gender, omitting the correspondence factor.

## Abbreviations

SNP: single nucleotide polymorphism
m5C: 5-methylcytosine
NSUN: NOL1/NOP2/sun
LD: linkage disequilibrium
HWE: Hardy-Weinberg equilibrium
OR: odds ratio
CI: confidence interval
m6A: N6-methyladenosine
