# Supplementary material for: Polymorphisms in the NSUN1 gene and neuroblastoma risk in Chinese children from Jiangsu province
Source: J Cancer. 2025 Jan 1;16(2):622–8. doi: 10.7150/jca.103097 (PMC11685692; doi:10.7150/jca.103097)
Supplement: Supplementary file 1 — Supplementary table. [file jcav16p0622s1.pdf]

Table S1. Demographic characteristics of neuroblastoma patients and cancer-free controls from Jiangsu province

Variables	Cases (N=402)		Controls (N=473)		P [a]
	No.	%	No.	%	
Age range, month	0.033-168.00		0.367-168.00		
Mean ± SD	40.99 ± 35.49		40.88 ± 29.76		0.962 [b]
Age					0.100
≤18 months	139	34.58	139	29.39	
>18 months	263	65.42	334	70.61	
Gender					0.987
Female	191	47.51	225	47.57	
Male	211	52.49	248	52.43	
Sites of origin					
Adrenal gland	93	23.13	/	/	
Retroperitoneal region	167	41.54	/	/	
Mediastinum	120	29.85	/	/	
Other region	18	4.48	/	/	
NA	4	1.00	/	/	
INSS stages					
I	108	26.87	/	/	
II	63	15.67	/	/	
III	59	14.68	/	/	
IV	104	25.87	/	/	
4s	2	0.50	/	/	
NA	66	16.42	/	/	

SD, standard deviation; NA, not available; INSS, International Staging System for Neuroblastoma.

[a] Two-sided χ^2 test between neuroblastoma patients and cancer-free controls.

[b] t test between neuroblastoma patients and cancer-free controls.
